# Revealing Details in Light and Shadows

**DOI:** 10.3201/eid2405.AC2405

**Published:** 2018-05

**Authors:** Byron Breedlove, Reginald Tucker

**Affiliations:** Centers for Disease Control and Prevention, Atlanta, Georgia, USA

**Keywords:** art science connection, emerging infectious diseases, art and medicine, about the cover, Kobayashi Kiyochika, Mosquito Net and Full Moon at Shinagawa, revealing details in light and shadows, mosquito-borne diseases, vector-borne infections, public health

**Figure:**
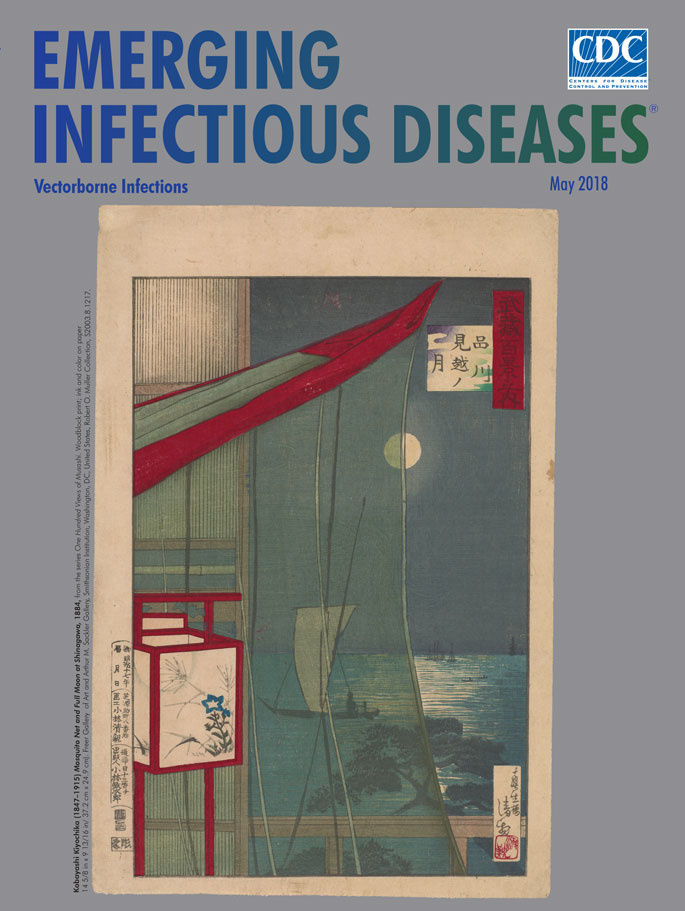
**Kobayashi Kiyochika (1847–1915), Mosquito Net and Full Moon at Shinagawa, from the series One Hundred Views of Musashi.** Woodblock print; ink and color on paper, 14 5/8 in x 9 13/16 in/ 37.2 cm x 24.9 cm. Freer Gallery of Art and Arthur M. Sackler Gallery, Smithsonian Institution, Washington, DC, United States, Robert O. Muller Collection, S2003.8.1217.

During the Edo period (1603–1868) in Japan, a time when Tokugawa Ieyasu and his descendants ruled, economic growth gave rise to a more literate, sophisticated, and affluent culture, hungry for the trappings of luxury, including extravagant art. This cultural shift was described by the phrase ukiyo, or “floating world,” which was associated with the pursuit of pleasure and hedonism in urban areas. The art of the Edo period, dominated by painting and woodblock prints, came to be known as “pictures of the floating world” or ukiyo-e.

As an artist who successfully fused motifs and ideas from Japanese and Western art, Kobayashi Kiyochika occupies a unique niche among Japanese illustrators of the Edo period. Japanese art historian, dealer, and collector Richard Lane considers Kiyochika to be both the last important ukiyo-e artist and the first exponent of the modern Japanese woodcut. Kato Yosuke, curator of Nerima Art Museum, Tokyo, explains that “Kiyochika is often referred to as the last ukiyo-e artist” because he stuck to colored woodblock prints and “kept pinning his hope on their potential until the end, despite the diversification and development of printing techniques in modern times.”

Kiyochika’s ukiyo-e color woodblock prints, as well as his newspaper illustrations and wartime propaganda art, document the rapid modernization that occurred during the reign of Emperor Meiji (1867–1912). As Japan transitioned from being an isolated shogunate state to becoming an imperial world power, it experienced an industrial revolution and opened its ports and cities to other countries. Yosuke wrote that Kiyochika “must have had the pride of a defeated person because he was a vassal of the shōgun born in Edo (present-day Tokyo).”

Miriam Wattles, professor of art at the University of California Santa Barbara, who specializes in Japanese visual art, sees distinctive influences from both Western and Japanese cultures in Kiyochika’s prints. Wattles states that his series of *One Hundred Views of Musashi*, which includes this month’s cover image, *Mosquito Net and Full Moon at Shinagawa*, “appropriates perspective, format, and style from Katsushika Hokusai, the most prolific and recognized Japanese artist of the late Edo period.” Wattles and others suggest that English painter Charles Wirgman, who in 1861 went to Japan on assignment as the visual reporter for *London Illustrated News*, influenced and may have briefly instructed the print maker.

This print displays the artist’s mastery of kōsen-ga, or “pictures of sunbeams,” a technique that portrays the interplay of light and shadows. *Mosquito Net and Full Moon at* *Shinagawa* appears a deviously simple subject, but Kiyochika’s composition, rendered in a photographic perspective, is brimming with details and contrasts that invite close scrutiny and calmness.

The edge of the draped mosquito net bisects a full moon; its mesh diffuses the moonlight and slices the moon into a yin and yang of darkness and light. The shimmering moonlight reflects on the surface of the water, and the artist contrasts not only the interplay of light and dark, but offers two views: one unobstructed, one through the netting. While several smaller boats drift near the horizon, Kiyochika placed a solitary sailboat near the center of the print, passing across the view and visible through the netting. Carefully etched vertical lines define the bamboo structure and contrast with the horizontal rippling shadows and reflections of the water’s surface. A jutting tree limb reaches over the water, yet no land is visible. There is a tactile texture, too. It’s easy to imagine the tautness of a paper lantern, the washboard surface of a bamboo wall, or the feel of a gauzy mesh mosquito net.

Although mosquito nets were introduced to Japan from China as early as 720ace, during the Edo period, silken nets were desired as luxury items and were widely used well into the 20th century, until glass windows and doors, air conditioning, and pesticides became commonplace. Mosquito control with long-lasting insecticidal nets continues to offer simple, inexpensive, but not infallible protection to people in many parts of the world. However, a confluence of demographic, environmental, and societal factors are enabling mosquitos and other vectors that spread disease-causing pathogens to expand their territories. Developing diverse integrated, innovative approaches to use alongside those measures that remain effective and to replace those that are no longer effective at controlling the spread of vectorborne diseases remains a critical public health imperative.
